# On the conditional performance of the synthetic chart with unknown process parameters using the exceedance probability criterion

**DOI:** 10.1371/journal.pone.0239538

**Published:** 2020-10-05

**Authors:** XueLong Hu, AnAn Tang, YuLong Qiao, JinSheng Sun, BaoCai Guo

**Affiliations:** 1 School of Management, Nanjing University of Posts and Telecommunications, Nanjing, China; 2 Institute of High-Quality Development Evaluation, Nanjing University of Posts and Telecommunications, Nanjing, China; 3 School of Automation, Nanjing University of Science and Technology, Nanjing, China; 4 School of Statistics and Mathematics, Zhejiang Gongshang University, Zhejiang, China; Universita degli Studi di Milano, ITALY

## Abstract

Recent researches on the control charts with unknown process parameters have noticed the large variability in the conditional in-control average run length (*ARL*) performance of control charts, especially when a small number of Phase I samples is used to estimate the process parameters. Some research works have been conducted on the conditional *ARL* performance of different types of control charts. In this paper, by simulating the empirical distribution of the conditional *ARL* and especially using the exceedance probability criterion (*EPC*), we study the conditional *ARL* performance of the synthetic $\bar{X}$ chart. Our results show that a large amount of Phase I samples is needed to obtain a specified *EPC* of the synthetic chart. For the available number of Phase I samples, the control limits of the synthetic chart are adjusted using the *EPC* method to improve its conditional in-control performance. It is shown that, for small mean shift sizes, a tradeoff should be made between the conditional in-control and out-of-control performances. For moderate to large shifts, the conditional performance of the synthetic chart using the adjusted control limits is generally satisfied. By comparing the results with the ones using the bootstrap approach, it can also be concluded that the conditional performances of both approaches are comparable. While the method proposed in this paper requires much less computation work than the bootstrap approach.

## 1 Introduction

Control charts are one of the primary techniques used in statistical process control (SPC) to monitor and detect changes in the process. They help engineers to bring back the process to a stable state. The Shewhart $\bar{X}$ chart has been widely implemented in practice by plotting the values of the sample statistic on a chart with upper (*UCL*) and lower (*LCL*) control limits (Aslam et al. [1]). The traditional Shewhart $\bar{X}$ chart is known to be effective in detecting large shift sizes in the process mean. While for small to moderate mean shift sizes, it is not as good as other type charts, like for instance, the Exponentially Weighted Moving Average (EWMA) chart, the Cumulative Sum (CUSUM) chart or the synthetic chart proposed in Wu and Spedding [2], which has drawn much attention in recent years. As it has been shown in Wu and Spedding [2] and Knoth [3], in the zero-state mode, the synthetic chart with optimal parameters performed better than the Shewhart chart for the detection of a small shift. Compared with the EWMA chart, the synthetic chart was better only for a particular large shift and for other shifts, the EWMA chart was uniformly better than the synthetic chart. In the steady-state mode, the EWMA chart uniformly performed better than the synthetic chart. Despite the disadvantage of the synthetic chart compared with the EWMA type chart, the related area still received much attention. This may be attributed to the fact that the practitioners may prefer waiting until the occurrence of a second sample beyond the control limits, before looking for an assignable cause. Moreover, according to Shongwe and Graham [4], there were other types of synthetic charts and some of them had better performance than the traditional one considered in this paper. Consequently, more research works were conducted on the synthetic charts.

Following the work of Wu and Spedding [2], Davis and Woodall [5] investigated the performance of the synthetic $\bar{X}$ chart using a Markov chain method. This method made it easier to study the theoretical run length (*RL*) properties of the synthetic chart. Khoo et al. [6] proposed a synthetic double sampling (DS) chart that combines the DS $\bar{X}$ and the conforming run length (CRL) charts. It was shown that the synthetic DS chart outperforms both the synthetic and the DS $\bar{X}$ charts. Based on the median run length (*MRL*) metric, Khoo et al. [7] evaluated the optimal performance of the synthetic $\bar{X}$ chart under the zero- and steady-state modes. Yen et al. [8] proposed three synthetic-type control charts to monitor the mean time-between-events of a homogenous Poisson process using a Markov chain approach. Both the zero- and steady-state average number of observations to signal (ANOS) were obtained to evaluate the performance of the three charts. More recently, Hu et al. [9] studied the economic performance of the synthetic chart for monitoring the process variance by taking measurement errors into account. When the process parameters are estimated, Lee et al. [10] evaluated the economic-statistical performance of the synthetic *np* chart. Celano and Castagliola [11] proposed a synthetic chart monitoring the ratio of two normal random variables. The statistical performance of the chart for known and random shift sizes were investigated in detail. To this end, it can be seen that synthetic type charts have received and continue to receive a lot of interest from researchers. For a comprehensive literature review on synthetic type charts, readers may refer to Rakitzis et al. [12].

Most research works on control charts usually assume that the process parameters used to construct the control limits are known. While in practice, the process parameters are usually unknown and they have to be estimated from a Phase I dataset composed of *m* initial samples, each of size *n*. This stage is called Phase I. Then in Phase II, samples from the process are collected and plotted on the control chart for the process monitoring. The properties of a control chart in Phase II varies from different practitioners because an inherent variability exists in the process parameters estimated from different Phase I dataset. This is recently called the between-practitioners variability in control charts (see Saleh et al. [13]). Chakraborti [14] was among the first group of researchers to emphasize the inherent variability in the conditional false alarm rate (*CFAR*). Then Epprecht et al. [15] studied the *CFAR* performance of the *S*^2^ and *S* charts and made some recommendations on the amount of Phase I samples to guarantee that the *CFAR* not exceed the specified upper prediction bound with a high probability. This was the exceedance probability criterion (*EPC*) introduced in Albers et al. [16]. To evaluate a control chart’s performance, the average run length (*ARL*), defined as the average number of samples until the chart gives a signal, is usually suggested. Similar to the *EPC* in Epprecht et al. [15] and Albers et al. [16], in this paper, we set a lower prediction bound on the *conditional* in-control *ARL* (denoted as *CARL*_*in*_) and make some recommendations on the amount of Phase I samples to guarantee that the *CARL*_*in*_ exceed the specified in-control *ARL* (denoted as *ARL*_0_) with a high probability 1 − *α*.

Accounting for the between-practitioners variability in the *ARL* performance of control charts, the standard deviation of *ARL* (*SDARL*) was also suggested recently to make recommendations on the amount of Phase I dataset to get the chart’s performance close to the one with known process parameters, see for instance, Zhang et al. [17], Jones and Steiner [18], Saleh et al. [19], Aly et al. [20], Faraz et al. [21] and Aly et al. [22]. They all pointed out that there is practically not enough Phase I dataset available to get a small *SDARL* value of control charts, which means that the conditional *ARL* (*CARL*) performance of a specific control chart may vary widely from the desired *ARL*. Readers may refer to Jensen et al. [23], Psarakis et al. [24] and therein for a comprehensive literature review on the effect of parameter estimations on the *ARL* performance of control charts.

When between-practitioners variability exists, to guarantee the *CARL*_*in*_ performance of control charts, the bootstrap method was recommended by Gandy and Kvaløy [25] to adjust the control limits such that a large probability of control charts have *CARL*_*in*_ larger than *ARL*_0_. Following this work, the bootstrap approach was applied to different type of control charts to adjust the control limits, see Faraz et al. [21] for the Shewhart *S*^2^ chart, Saleh et al. [13] for the EWMA $\bar{X}$ chart, Aly et al. [20] for the adaptive EWMA $\bar{X}$ chart, Hu et al. [26] for the Shewhart median chart and so on. However, applying the bootstrap approach to adjust the control limits is computationally intensive. Some other researchers recommended the analytical method to adjust the control limits. Goedhart et al. [27] investigated the conditional properties of the Shewhart $\bar{X}$ chart and showed that both the analytical and the bootstrap approaches get similar performances. The analytical approach was also extended to the Shewhart *S*^2^ chart to adjust the control limits, see Faraz et al. [21] and Goedhart et al. [28]. It is noticed that the analytical approach was only applied to the Shewhart type chart, where the chart statistics are independent. For the EWMA or CUSUM type charts, only the bootstrap approaches were used to adjust the control limits, see Saleh et al. [13] and Saleh et al. [29]. Moreover, Hu et al. [30] investigated the *CARL* performance of the synthetic $\bar{X}$ chart using the *SDARL* criteria and they also applied the bootstrap approach to adjust the control limits. For more discussions of the synthetic type charts with estimated parameters, readers may refer to Zhang et al. [31], Castagliola et al. [32], Guo and Wang [33] and You et al. [34]. Differing from the work of Hu et al. [30], these researches were focused on the marginal (unconditional) performance of the synthetic type chart.

Since the bootstrap approach to adjust the control limits of control charts is time consuming, an approach that using an empirical distribution to approximate the *CARL*_*in*_ distribution and then to get the adjusted control limits of the EWMA $\bar{X}$ chart was suggested in Diko et al. [35]. Motivated by this work, we study the *CARL* properties of the synthetic $\bar{X}$ chart under the zero-state mode using the *EPC* and adjust the control limits using the empirical distribution of *CARL*_*in*_. It is important to note that this paper is not to show the superiority of the synthetic chart but to investigate the conditional performance of the synthetic chart using the EPC approach. In addition, similar methods can be applied to study the steady-state properties of the synthetic chart. The remainder of this paper is structured as follows: the synthetic $\bar{X}$ chart with unknown process parameters is presented in Section 2. Then Section 3 evaluates the *CARL* performance of the synthetic $\bar{X}$ chart using the *EPC* and makes some recommendations on the number of Phase I samples to get the desired conditional performance of the chart. Using the simulated empirical distribution of the *CARL* and the *EPC*, Section 4 presents the adjusted control limits of the synthetic $\bar{X}$ chart to guarantee the *CARL* values exceed the desired *ARL*_0_ with a specified high probability and the *CARL* performance of the synthetic $\bar{X}$ chart using the unadjusted and adjusted control limits are investigated in Section 5. Moreover, comparisons are also made with the bootstrap approach in Hu et al. [30]. Finally, some conclusions and recommendations are given in Section 6.

## 2 Synthetic $\bar{X}$ chart with unknown process parameters

When the process parameters *μ*_0_ and *σ*_0_ are unknown, the mean estimator ${\hat{\mu }}_{0}=\frac{1}{m}\sum_{i=1}^{m}{\bar{X}}_{i}$ and the standard deviation estimator ${\hat{\sigma }}_{0}=\sqrt{\frac{1}{m(n-1)}\sum_{i=1}^{m}\sum_{j=1}^{n}{\left(X_{i,j}-{\bar{X}}_{i}\right)}^{2}}$ are usually suggested, where {*X*_*i*,1_, …, *X*_*i*, *n*_}, *i* = 1, 2, …, *m* are assumed to be independently normally distributed Phase I samples with unknown mean ${\hat{\mu }}_{0}$ and standard deviation ${\hat{\sigma }}_{0}$ and ${\bar{X}}_{i}=\frac{1}{n}\sum_{j=1}^{n}X_{i,j}$ is the *i*th sample mean (see Jensen et al. [23], Schoonhoven et al. [36]).

In Phase II, Let {*Y*_*i*,1_, …, *Y*_*i*, *n*_}, *i* = 1, 2, …, be a sample of size *n* ≥ 1 from an independent normal random variables, i.e., *Y*_*i*, *j*_ ∼ *N*(*μ*_0_+ *δσ*_0_, *σ*_0_), *j* = 1, 2, …, *n*, where *μ*_0_ and *σ*_0_ are the actual in-control mean and standard deviation, respectively. *δ* is the size of the standardized mean shift. If *δ* = 0, the process is in-control. Otherwise, the process is out-of-control. The sample mean ${\bar{Y}}_{i}=\frac{1}{n}\sum_{j=1}^{n}Y_{i,j}$ is plotted on the chart for the Phase II process monitoring.

For the synthetic $\bar{X}$ chart, two sub-charts: an $\bar{X}$ sub-chart and a CRL sub-chart are combined together. A sample is considered as a nonconforming one on the $\bar{X}$ sub-chart if ${\bar{Y}}_{i}$, *i* = 1, 2, …, falls outside the predetermined control limits (see Eqs (1) and (2) below). Then the number of inspected units between two most recent consecutive nonconforming units inclusive of the nonconforming unit at the end, is counted and denoted as a CRL value. The state of the process is decided by the comparison of the control limit *H* of the CRL sub-chart and the CRL value. The procedures of the synthetic $\bar{X}$ chart are listed as follows:

(1) Specify the sample size *n*, the desired *ARL*_0_ and the standardized mean shift size *δ*.
(2) Set up the control limits $\hat{LCL}$ and $\hat{UCL}$ of the $\bar{X}$ sub-chart as follows:
  $$\begin{array}{c} \hat{LCL}={\hat{\mu }}_{0}-K{\hat{\sigma }}_{0}, \end{array}$$ (1)
  $$\begin{array}{c} \hat{UCL}={\hat{\mu }}_{0}+K{\hat{\sigma }}_{0}, \end{array}$$ (2)
  where *K* > 0 is the control limit coefficient of the $\bar{X}$ sub-chart.
(3) Take a sample {*Y*_*i*,1_, …, *Y*_*i*, *n*_} at each sampling point *i* = 1, 2, … and compute the sample mean ${\bar{Y}}_{i}$.
(4) If ${\bar{Y}}_{i}\in \left[LCL,UCL\right]$ (see Eqs (1) and (2)), the control flow goes back to step (3) to take the next sample; Otherwise, ${\bar{Y}}_{i}\notin \left[LCL,UCL\right]$, the control flow goes to the next step.
(5) Count the *CRL* value. If *CRL* > *H*, the process is considered to be in-control and the control flow goes back to step (4). Otherwise, the process is declared as out-of-control and the control flow goes to the next step.
(6) An out-of-control signal is given to indicate a potential process shift.

Performance of a control chart is usually evaluated by its *RL* properties. For a Phase I dataset, the *RL* properties of the chart is conditioned on the estimated process parameters ${\hat{\mu }}_{0}$ and ${\hat{\sigma }}_{0}$. In order to obtain the *CARL* of the synthetic $\bar{X}$ chart with unknown process parameters, a Markov chain method in Davis and Woodall [5] is used to model the chart and the transition probability matrix **P**_(*H*+2)×(*H*+2)_ in the Markov chain is given by,

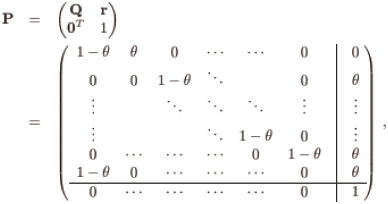
(3)
where **Q**_(*H*+1)×(*H*+1)_ is a transition probability matrix for the transient states, $0_{1\times (H+1)}^{T}=\left(0,0,\ldots ,0\right)$ is a row vector, the column vector **r**_(*H*+1)×1_ satisfies **r** = **1** − **Q1** with **1** = (1, 1, …, 1)^*T*^. Moreover, $\theta =P({\bar{Y}}_{i}\notin \left[\hat{LCL},\hat{UCL}\right])$ = $P\left({\bar{Y}}_{i}\leq \hat{LCL}\right)+P\left({\bar{Y}}_{i}\geq \hat{UCL}\right)$ is the probability of a nonconforming sample on the $\bar{X}$ sub-chart and can be given as,

$$\begin{array}{c} \theta =F_{N}(\left({\hat{\mu }}_{0}-K{\hat{\sigma }}_{0}-\mu_{0}-\delta \sigma_{0}\right)\frac{\sqrt{n}}{\sigma_{0}})+1\\ -F_{N}(\left({\hat{\mu }}_{0}+K{\hat{\sigma }}_{0}-\mu_{0}-\delta \sigma_{0}\right)\frac{\sqrt{n}}{\sigma_{0}}), \end{array}$$ (4)

where *F*_*N*_(⋅) is the cumulative distribution function (c.d.f.) of the standard normal distribution. More information on the derivation of Eq (4) can be found in Zhang et al. [31]. Then the *CARL* of the synthetic $\bar{X}$ chart, conditioned on given values of ${\hat{\mu }}_{0}$ and ${\hat{\sigma }}_{0}$ can be obtained as (see Neuts [37] or Latouche and Ramaswami [38]),

$$\begin{array}{c} CARL=q^{T}{\left(I-Q\right)}^{-1}1, \end{array}$$ (5)

where the initial probabilities **q** = (0, 1, 0, …, 0)^*T*^ and **I**_(*H*+1)×(*H*+1)_ is an identity matrix.

Define the random variables $U=\left({\hat{\mu }}_{0}-\mu_{0}\right)\frac{\sqrt{n}}{\sigma_{0}}$ and $V=\frac{{\hat{\sigma }}_{0}}{\sigma_{0}}\sqrt{n}$, then the variable *U* follows a normal distribution, i.e., $f_{U}\left(u|m\right)=f_{N}(u|0,\frac{1}{\sqrt{m}})$, where *f*_*N*_(⋅) is the p.d.f. of a normal distribution. According to Zhang et al. [31], *V*^2^ follows a gamma $\gamma (\frac{m(n-1)}{2},\frac{2n}{m(n-1)})$ distribution and the p.d.f. of the variable *V* is deduced as $f_{V}\left(v|m,n\right)=2vf_{\gamma }(v^{2}|\frac{m(n-1)}{2},\frac{2n}{m(n-1)})$, where *f*_*γ*_(⋅) is the p.d.f. of the gamma distribution with parameters $\frac{m(n-1)}{2}$ and $\frac{2n}{m(n-1)}$. Since random variables *U* and *V* are independent, then the unconditional or the average *ARL* (*AARL*) of the synthetic $\bar{X}$ chart with estimated parameters is,

$$\begin{array}{ccc} AARL & = & E(CARL)\\ & = & \int_{-\infty }^{+\infty }\int_{0}^{+\infty }CARL\times f_{U}\left(u|m\right)f_{V}\left(v|m,n\right)dvdu, \end{array}$$

and the standard deviation of *ARL* (*SDARL*) is

$$\begin{array}{c} SDARL=\sqrt{E\left(CARL^{2}\right)-{\left(E\left(CARL\right)\right)}^{2}}, \end{array}$$ (6)

where

$$\begin{array}{ccc} & & E(CARL^{2})\\ & & =\int_{-\infty }^{+\infty }\int_{0}^{+\infty }CARL^{2}\times f_{U}\left(u|m\right)f_{V}\left(v|m,n\right)dvdu. \end{array}$$

For fixed values of *H*, *K*, *m*, *n* and *δ*, it is noted that the *CARL* is a random variable depending on ${\hat{\mu }}_{0}$ and ${\hat{\sigma }}_{0}$. When the process is in-control (*δ* = 0), Hu et al. [30] studied the performance of the chart using Eq (6) and pointed out that the *CARL*_*in*_ values generally vary widely from the desired *ARL*_0_ if using the control chart parameters designed in the known process parameters case. This causes much more false alarms than expected and surely calls into question the process monitoring regime. Hence recent literature on the conditional properties of control charts with unknown process parameters is designed to guarantee,

$$\begin{array}{c} P(CARL_{in}>ARL_{0})=1-\alpha , \end{array}$$ (7)

where *α* ∈ (0, 1) is a constant. It can be seen that the desired *ARL*_0_ is the 100*α*th percentile of the distribution of *CARL*_*in*_. The standard deviation of *CARL*_*in*_ (*SDCARL*_*in*_) used in Hu et al. [30] only accounted for the variability in the *CARL*_*in*_ values, and it is still different from the *EPC* method in Eq (7) which also considers the shape and skewness of the *CARL*_*in*_ distribution. By using this *EPC* approach, we evaluate the properties of the synthetic $\bar{X}$ chart in the next sections.

## 3 Performance assessment of the synthetic $\bar{X}$ chart with unknown process parameters

It is noted that the *EPC* approach in Eq (7) can also be re-written as,

$$\begin{array}{c} P(CARL_{in}\leq ARL_{0})=\alpha . \end{array}$$ (8)

In Eq (8), it is desired that the 100*α*th percentile, denoted as *CARL*_*in*,*α*_, of the *CARL*_*in*_ distribution is equal to *ARL*_0_. While using the control limits designed in the known process parameters case, the value of *CARL*_*in*,*α*_ is generally smaller than *ARL*_0_ (see Table 1). We are now wondering that how large the difference between *CARL*_*in*,*α*_ and the desired *ARL*_0_ is when *m* Phase I samples are used to estimate the process parameters? The percentage difference between *CARL*_*in*,*α*_ and *ARL*_0_, defined as *PD* = (*CARL*_*in*,*α*_ − *ARL*_0_)/*ARL*_0_ × 100%, is used to evaluate the conditional performance of the synthetic $\bar{X}$ chart. It should be explained that a negative *PD* means that *CARL*_*in*,*α*_ < *ARL*_0_ by *PD* percentage points and a positive *PD* means that *CARL*_*in*,*α*_ > *ARL*_0_ by *PD* percentage points. For different values of *ARL*_0_ ∈ {200, 370.4, 500}, *α* ∈ {0.05, 0.10}, (*H*, *K*) and *m*, Table 1 presents the *CARL*_*in*,*α*_ values. For simplicity, the corresponding *PD* values are not shown in the table. The parameters (*H*, *K*) of the synthetic $\bar{X}$ chart used here are optimized for different shift sizes *δ* ∈ {0.2, 0.4, 0.6, 0.8, 1.0, 1.4} and the desired *ARL*_0_ when the process parameters are *known*. From Table 1, several conclusions are made as follows:

- For a small value of *m*, the *CARL*_*in*,*α*_ is small and the absolute value of *PD* is large. For example, when *ARL*_0_ = 200, *α* = 0.1, (*δ*, *H*, *K*) = (0.2, 41, 1.1177) and *m* = 50, *CARL*_*in*,*α*_ = 82.68 and the absolute value of *PD* is 58.66%. This means that the *CARL*_*in*_ value of the synthetic chart is larger than 82.68 with 90% probability. The value *CARL*_*in*,*α*_ = 82.68 is practically much smaller than the desired *ARL*_0_ = 200, which may cause much more false alarms in the process monitoring. With the increase in the value of *m*, the value of *CARL*_*in*,*α*_ increases and the absolute value of *PD* decreases. For example, for the same case presented above but with *m* = 10^4^, the *CARL*_*in*,*α*_ = 189.02 is much larger than 82.68 and is close to the desired *ARL*_0_, and the absolute value of *PD* is 5.49%, which is much smaller than 58.66%.
- For fixed values of *ARL*_0_, *m* and *α*, when the anticipated shift size *δ* increases (or the value of *H* decreases), the value of *CARL*_*in*,*α*_ increases and the absolute value of *PD* decreases. For example, when *ARL*_0_ = 200, *α* = 0.1 and *m* = 50, the value of *δ* increases from 0.2 up to 1.4 (or the value of *H* decreases from 41 down to 2), the value of *CARL*_*in*,*α*_ increases from 82.68 up to 102.33 and the absolute value of *PD* decreases from 58.66% down to 48.83%. Since the Shewhart $\bar{X}$ chart is a special case of the synthetic $\bar{X}$ chart with *H* = + ∞, we can also conclude that the conditional in-control performance of the synthetic chart is better than the one of the Shewhart $\bar{X}$ chart.
- For fixed values of *ARL*_0_, *m* and *δ*, it can be seen that larger values of *α* are associated with larger *CARL*_*in*,*α*_ values and smaller absolute values of *PD*. For example, when *ARL*_0_ = 200, *m* = 50 and *δ* = 0.2, the values of *CARL*_*in*,*α*_ are 67.83 and 82.68, and the absolute values of *PD* are 66.06% and 58.66% corresponding to *α* = 0.05 and *α* = 0.1, respectively. This is explained by the fact that a large value of *α* means a smaller probability to get the *CARL*_*in*_ values larger than *ARL*_0_.

**Table 1:** The 5th and 10th percentiles of the *CARL*_*in*_ values of the synthetic $\bar{X}$ chart for different values of *ARL*_0_, *m* and (*H*, *K*) when *n* = 5.

	*α* = 0.05	*α* = 0.1	*α* = 0.05	*α* = 0.1	*α* = 0.05	*α* = 0.1	*α* = 0.05	*α* = 0.1	*α* = 0.05	*α* = 0.1	*α* = 0.05	*α* = 0.1
*m*	*ARL*_0_ = 200											
	(*δ*, *H*, *K*) = (0.2, 41, 1.1177)		(*δ*, *H*, *K*) = (0.4, 17, 1.0540)		(*δ*, *H*, *K*) = (0.6, 8, 0.9945)		(*δ*, *H*, *K*) = (0.8, 5, 0.9553)		(*δ*, *H*, *K*) = (1.0, 3, 0.9108)		(*δ*, *H*, *K*) = (1.4, 2, 0.8741)	
50	67.83	82.68	70.52	85.52	76.34	89.95	78.58	93.07	84.19	99.99	88.92	102.33
100	92.66	108.18	96.75	111.39	100.05	116.62	104.63	120.99	109.63	124.56	114.04	127.04
200	118.25	130.60	113.20	134.12	125.45	137.27	128.87	141.32	131.84	143.66	134.89	147.43
300	130.47	141.98	133.47	143.99	135.96	148.33	139.86	149.51	143.29	153.09	146.57	154.81
400	137.24	148.27	140.67	151.20	143.81	154.97	146.25	156.19	148.95	159.54	152.56	160.48
500	143.59	154.27	146.26	156.00	149.66	158.95	152.44	160.79	154.73	162.97	157.32,	166.02
600	148.43	157.50	149.97	158.64	153.17	160.84	155.33	164.65	157.89	166.61	159.86	167.36
700	151.46	160.81	154.11	161.86	156.82	164.99	158.36	166.79	160.83	168.95	163.41	170.47
800	153.56	162.73	156.63	164.80	158.97	167.02	161.00	168.34	163.90	170.65	165.53	172.17
1000	159.00	166.39	161.18	167.85	162.84	170.40	165.01	171.21	167.13	173.49	168.62	175.34
1500	165.84	172.48	167.53	174.04	168.80	175.69	170.19	176.82	172.18	178.12	174.52	179.74
2000	169.63	175.88	171.48	176.68	173.39	179.06	174.45	180.11	176.34	181.34	177.83	181.95
2500	172.62	178.29	173.71	179.59	175.90	180.52	177.30	181.91	178.86	183.12	179.72	184.40
5000	180.38	184.21	181.26	185.40	182.91	186.23	183.59	187.05	184.95	187.92	185.69	188.70
7500	183.76	187.00	184.77	187.78	185.92	188.76	186.13	189.20	187.52	190.04	188.21	190.74
10000	185.88	189.02	186.66	189.23	187.68	190.26	188.44	190.84	188.96	191.42	189.72	191.91
	*ARL*_0_ = 370.4											
	(*δ*, *H*, *K*) = (0.2, 60, 1.1966)		(*δ*, *H*, *K*) = (0.4, 23, 1.1297)		(*δ*, *H*, *K*) = (0.6, 11, 1.0741)		(*δ*, *H*, *K*) = (0.8, 6, 1.0259)		(*δ*, *H*, *K*) = (1.0, 4, 0.9923)		(*δ*, *H*, *K*) = (1.4, 2, 0.9324)	
50	107.78	134.46	113.78	142.55	120.85	148.50	129.61	157.52	136.46	167.23	150.29	177.21
100	156.05	183.67	162.25	192.85	169.15	199.46	176.66	206.60	184.58	212.72	196.42	220.78
200	202.26	229.39	208.76	232.76	215.28	241.46	223.97	246.21	230.73	253.79	240.12	261.13
300	223.95	250.52	232.73	254.26	240.72	260.90	248.91	268.68	252.57	271.43	259.24	279.69
400	243.53	264.41	246.73	267.40	254.52	275.05	259.37	280.23	263.24	284.76	274.91	291.05
500	255.55	277.01	257.53	278.56	264.60	283.45	268.19	289.68	274.68	293.49	281.33	299.54
600	261.39	281.45	267.40	283.77	272.03	290.80	278.21	295.33	282.56	299.46	289.64	305.74
700	268.37	286.61	272.45	291.81	278.21	296.15	284.90	300.74	287.06	303.88	295.63	309.35
800	273.77	293.39	279.52	296.99	283.96	301.12	289.44	304.60	292.76	307.03	300.51	313.12
1000	284.66	300.15	286.66	303.02	292.94	307.89	297.53	309.99	300.50	314.08	306.83	319.47
1500	298.00	311.32	300.99	315.05	305.24	318.60	308.82	321.23	312.72	322.50	317.78	328.20
2000	306.77	319.94	309.33	322.55	314.08	324.73	315.67	326.79	320.46	329.56	323.30	333.68
2500	312.82	323.90	314.74	326.70	320.31	329.16	323.20	332.08	325.50	333.45	329.18	337.36
5000	328.16	336.91	330.45	339.16	333.17	341.62	335.94	343.06	337.90	344.64	340.71	346.95
7500	337.32	343.16	337.78	344.75	340.55	346.81	342.23	347.77	343.50	349.12	346.21	351.17
10000	340.55	347.27	342.85	348.03	344.63	350.08	345.18,	351.04	347.11	351.36	349.07	353.62
	*ARL*_0_ = 500											
	(*δ*, *H*, *K*) = (0.2, 73, 1.2341)		(*δ*, *H*, *K*) = (0.4, 26, 1.1634)		(*δ*, *H*, *K*) = (0.6, 12, 1.1061)		(*δ*, *H*, *K*) = (0.8, 6, 1.0517)		(*δ*, *H*, *K*) = (1.0, 4, 1.0186)		(*δ*, *H*, *K*) = (1.4, 2, 0.9597)	
50	133.33	167.89	144.45	179.05	151.82	189.26	165.49	204.89	173.74	208.77	190.82	227.51
100	196.41	238.46	206.30	245.76	223.58	258.29	229.36	267.46	239.44	280.46	257.59	291.68
200	264.55	299.43	267.31	306.84	280.97	318.02	292.66	325.56	301.04	335.33	311.84	345.88
300	296.44	331.35	305.49	336.51	313.73	345.63	325.26	354.95	333.29	361.85	344.71	371.46
400	316.66	349.61	326.24	355.30	335.63	362.64	344.68	373.49	351.05	377.45	365.05	388.90
500	332.22	364.49	341.72	369.01	350.11	376.96	358.91	385.25	364.62	387.27	377.37	396.72
600	343.38	375.09	351.73	377.84	359.40	384.83	368.15	391.22	375.25	399.99	384.07	407.21
700	354.49	381.74	363.41	386.90	367.34	394.73	379.95	400.35	380.58	405.83	394.95	412.60
800	363.91	390.50	369.86	394.62	377.44	400.71	387.14	406.77	388.87	411.06	401.52	418.52
1000	377.37	398.76	381.77	405.30	390.23	411.54	395.07	415.14	402.42	419.94	409.74	428.25
1500	394.90	415.65	402.46	420.23	407.42	424.97	414.73	432.02	418.99	433.96	425.51	440.04
2000	410.52	428.14	415.18	429.04	419.56	437.24	426.19	439.89	428.59	443.50	435.25	447.48
2500	417.41	434.33	421.76	438.68	428.09	443.84	433.45	445.69	435.92	447.91	441.34	452.66
5000	439.70	452.48	444.58	455.87	448.29	459.22	450.89	461.54	453.90	463.22	457.75	466.62
7500	450.13	461.38	453.49	462.88	456.55	465.61	460.03	467.71	461.19	470.30	465.32	472.95
10000	457.57	466.36	458.48	468.20	461.83	469.89	465.02	472.37	467.06	474.06	470.06	476.47

With the available number of Phase I samples, readers can refer to Table 1 to predict the smaller *CARL*_*in*_ (compared with the desired *ARL*_0_) value of the synthetic $\bar{X}$ chart that can be attained with a probability 1−*α*. Some recommendations are also summarized in Table 2 on the roughly minimum number *m* of Phase I samples to guarantee the *CARL*_*in*_ values larger than *ARL*_0_ × (1 − *ϵ*) with a specified high probability 1 − *α*, where *ϵ* is a nominally specified value of *PD*. This is mathematically given as follows,

$$\begin{array}{c} P(CARL_{in}\geq ARL_{0}\times \left(1-\epsilon \right))\geq 1-\alpha , \end{array}$$ (9)

From the results in Table 2, it can be noted that, using the *EPC*, a large amount of Phase I samples is needed to get a quite satisfied *CARL*_*in*_ performance, especially when *ϵ* = 0% and *α* = 0.05. Even when *ϵ* = 20%, *α* = 0.1 and *ARL*_0_ = 200, the minimum number of Phase I samples needed to guarantee *CARL*_*in*_ ≥ *ARL*_0_ × (1 − 20%) with a high probability 90% is about *m* ≃ 400. In some applications with plenty data, it might be possible to accumulate up to *m* = 400 Phase I samples. But waiting for many more Phase I samples to estimate the process parameters is clearly unacceptable and can be detrimental for the quality of the product (see [39]). For the available number of Phase I samples in practice, using the control limits designed under known parameters cases can not guarantee the synthetic $\bar{X}$ chart’s *CARL*_*in*_ performance, the control limits of the chart should be adjusted to improve the chart’s conditional in-control performance.

**Table 2:** Minimum number *m* of Phase I samples to guarantee the *CARL*_*in*_ values larger than *ARL*_0_×(1 − *ϵ*) with a specified high probability 1 − *α*.

	*α* = 0.05	*α* = 0.1	*α* = 0.05	*α* = 0.1	*α* = 0.05	*α* = 0.1	*α* = 0.05	*α* = 0.1	*α* = 0.05	*α* = 0.1	*α* = 0.05	*α* = 0.1
*ϵ*	*ARL*_0_ = 200											
	(*δ*, *H*, *K*) = (0.2, 41, 1.1177)		(*δ*, *H*, *K*) = (0.4, 17, 1.0540)		(*δ*, *H*, *K*) = (0.6, 8, 0.9945)		(*δ*, *H*, *K*) = (0.8, 5, 0.9553)		(*δ*, *H*, *K*) = (1.0, 3, 0.9108)		(*δ*, *H*, *K*) = (1.4, 2, 0.8741)	
0%	>10000											
10%	4750	2950	4150	2650	3700	2300	3300	2050	2750	1800	2500	1550
20%	1100	700	1000	650	900	550	750	500	700	450	600	400
	*ARL*_0_ = 370.4											
	(*δ*, *H*, *K*) = (0.2, 60, 1.1966)		(*δ*, *H*, *K*) = (0.4, 23, 1.1297)		(*δ*, *H*, *K*) = (0.6, 11, 1.0741)		(*δ*, *H*, *K*) = (0.8, 6, 1.0259)		(*δ*, *H*, *K*) = (1.0, 4, 0.9923)		(*δ*, *H*, *K*) = (1.4, 2, 0.9324)	
0%	>10000											
10%	6250	3950	5600	3500	4950	3050	4250	2600	3900	2400	3300	2000
20%	1400	900	1300	800	1100	700	1000	600	850	550	750	450
	*ARL*_0_ = 500											
	(*δ*, *H*, *K*) = (0.2, 73, 1.2341)		(*δ*, *H*, *K*) = (0.4, 26, 1.1634)		(*δ*, *H*, *K*) = (0.6, 12, 1.1061)		(*δ*, *H*, *K*) = (0.8, 6, 1.0517)		(*δ*, *H*, *K*) = (1.0, 4, 1.0186)		(*δ*, *H*, *K*) = (1.4, 2, 0.9597)	
0%	>10000											
10%	7200	4500	6350	3900	5600	3350	4700	2900	4350	2650	3500	2200
20%	1600	1000	1450	850	1300	800	1100	650	1000	600	850	500

## 4 Adjustment of the control limits of the synthetic $\bar{X}$ chart to guarantee the conditional performance

From Eq (9), for fixed values of *α* and *ϵ*, the control limits (*H*, *K*) of the synthetic $\bar{X}$ chart should be adjusted to guarantee that the c.d.f. of *CARL*_*in*_ at *ARL*_0_ × (1 − *ϵ*) is smaller than *α*. Since a closed-form expression of the c.d.f. of *CARL*_*in*_ is not available, the empirical distribution of *CARL*_*in*_ is simulated to approximate the *CARL*_*in*_ distribution. By doing so, we can find the adjusted control limits of the synthetic $\bar{X}$ chart to satisfy Eq (9). The algorithms of the adjustments of control limits are outlined as follows,

(1) For the specified values of *ARL*_0_ and the desired shift size *δ*, the unadjusted parameters (*H*, *K*) of the synthetic $\bar{X}$ chart are firstly optimized for *ARL*_0_ and *δ* when the process parameters are known.
(2) Fix the value of parameter *H* and simulate the empirical distribution of *CARL*_*in*_ for different values of *K* ranging from the optimized value in step (1) to infinity. The empirical distribution of *CARL*_*in*_ is obtained from 10^4^ simulated synthetic $\bar{X}$ chart.
(3) For each simulated empirical distribution of *CARL*_*in*_, the *CARL*_*in*,*α*_ is calculated and is compared with *ARL*_0_ × (1 − *ϵ*). The first *CARL*_*in*,*α*_ that is equal or larger than *ARL*_0_ × (1 − *ϵ*) is recorded and the corresponding value of *K* is selected to be the adjusted parameter *K*_*a*_.

Using the above algorithms, we can find the adjusted control limits of the synthetic $\bar{X}$ chart. For different combinations of *ARL*_0_ ∈ {200, 370.4, 500}, *m* ∈ {50, 100, 150, 200, 205, 300, 350, 400} and *α* ∈ {0.05, 0.1}, when *ϵ* ∈ {0%, 10%, 20%}, Tables 3 to 5 present the adjusted parameter *K*_*a*_ that guarantee the values of *CARL*_*in*_ exceed the desired *ARL*_0_ with a specified high probability 1 − *α*. It should be noted that the unadjusted parameters (*H*, *K*) of the synthetic chart are optimized for different *δ* and *ARL*_0_ × (1 − *ϵ*) when the process parameters are known. From these tables, it can be noted that the adjusted control limits *K*_*a*_ are larger than the ones designed with known process parameters and the adjusted control limits decrease with the increase in *m* and converge to the value designed with known process parameters. For example, in Table 3, when *ARL*_0_ = 200, *α* = 0.05 and *δ* = 0.2, if *m* increases from 50 up to 400, the adjusted parameter decreases from *K*_*a*_ = 1.2324 down to *K*_*a*_ = 1.1524, which is more close to the *K* = 1.1177 designed with known process parameters. Moreover, with the increase in the value of *α* or *ϵ*, the adjusted control limits decrease. For example, when *ARL*_0_ = 200, *m* = 50 and *δ* = 0.2 (see Table 3), the adjusted parameter *K*_*a*_ decreases from 1.2324 down to 1.2080 when *α* increases from 0.05 up to 0.1. When *m* = 50, *δ* = 0.2, *α* = 0.1 and *ϵ* = 20%, we can also see from Table 5 that *K*_*a*_ = 1.1767, which is smaller than the corresponding *K*_*a*_ = 1.2080 in Table 3 when *ϵ* = 0%.

**Table 3:** The adjusted values of *K*_*a*_ that guarantee *P*(*CARL*_*in*_ > *ARL*_0_ × (1 − *ϵ*)) = 1 − *α* of the synthetic $\bar{X}$ chart for different values of *ARL*_0_, *m* and *α* when *n* = 5 and *ϵ* = 0%.

	*α* = 0.05	*α* = 0.1	*α* = 0.05	*α* = 0.1	*α* = 0.05	*α* = 0.1	*α* = 0.05	*α* = 0.1	*α* = 0.05	*α* = 0.1	*α* = 0.05	*α* = 0.1
*m*	*ARL*_0_ = 200											
	(*δ*, *H*, *K*) = (0.2, 41, 1.1177)		(*δ*, *H*, *K*) = (0.4, 17, 1.0540)		(*δ*, *H*, *K*) = (0.6, 8, 0.9945)		(*δ*, *H*, *K*) = (0.8, 5, 0.9553)		(*δ*, *H*, *K*) = (1.0, 3, 0.9108)		(*δ*, *H*, *K*) = (1.4, 2, 0.8741)	
50	1.2324	1.2080	1.1618	1.1384	1.0967	1.0751	1.0536	1.0328	1.0040	0.9850	0.9619	0.9446
100	1.1918	1.1768	1.1261	1.1106	1.0618	1.0477	1.0193	1.0062	0.9726	0.9595	0.9332	0.9205
150	1.1781	1.1648	1.1105	1.0984	1.0477	1.0369	1.0068	0.9959	0.9597	0.9497	0.9212	0.9115
200	1.1675	1.1581	1.1025	1.0919	1.0396	1.0302	0.9989	0.9897	0.9525	0.9433	0.9142	0.9055
250	1.1630	1.1532	1.0960	1.0878	1.0346	1.0258	0.9938	0.9855	0.9476	0.9398	0.9091	0.9018
300	1.1588	1.1498	1.0921	1.0840	1.0309	1.0234	0.9905	0.9828	0.9441	0.9368	0.9061	0.8989
350	1.1554	1.1474	1.0893	1.0818	1.0281	1.0210	0.9874	0.9803	0.9413	0.9348	0.9035	0.8975
400	1.1524	1.1452	1.0864	1.0798	1.0259	1.0191	0.9851	0.9783	0.9391	0.9331	0.9015	0.8955
	*ARL*_0_ = 370.4											
	(*δ*, *H*, *K*) = (0.2, 60, 1.1966)		(*δ*, *H*, *K*) = (0.4, 23, 1.1297)		(*δ*, *H*, *K*) = (0.6, 11, 1.0741)		(*δ*, *H*, *K*) = (0.8, 6, 1.0259)		(*δ*, *H*, *K*) = (1.0, 4, 0.9923)		(*δ*, *H*, *K*) = (1.4, 2, 0.9324)	
50	1.3180	1.2937	1.2449	1.2208	1.1841	1.1611	1.1317	1.1095	1.0941	1.0729	1.0282	1.0078
100	1.2774	1.2609	1.2062	1.1896	1.1474	1.1314	1.0956	1.0809	1.0602	1.0457	0.9948	0.9812
150	1.2603	1.2469	1.1900	1.1775	1.1313	1.1196	1.0809	1.0689	1.0450	1.0338	0.9824	0.9713
200	1.2516	1.2394	1.1813	1.1700	1.1236	1.1129	1.0726	1.0626	1.0370	1.0279	0.9749	0.9663
250	1.2446	1.2342	1.1750	1.1653	1.1175	1.1080	1.0671	1.0586	1.0322	1.0236	0.9699	0.9618
300	1.2405	1.2305	1.1715	1.1622	1.1130	1.1051	1.0632	1.0553	1.0288	1.0212	0.9669	0.9586
350	1.2365	1.2284	1.1672	1.1596	1.1103	1.1025	1.0602	1.0530	1.0256	1.0185	0.9636	0.9572
400	1.2341	1.2261	1.1652	1.1576	1.1072	1.1005	1.0576	1.0510	1.0232	1.0165	0.9615	0.9552
	*ARL*_0_ = 500											
	(*δ*, *H*, *K*) = (0.2, 73, 1.2341)		(*δ*, *H*, *K*) = (0.4, 26, 1.1634)		(*δ*, *H*, *K*) = (0.6, 12, 1.1061)		(*δ*, *H*, *K*) = (0.8, 6, 1.0517)		(*δ*, *H*, *K*) = (1.0, 4, 1.0186)		(*δ*, *H*, *K*) = (1.4, 2, 0.9597)	
50	1.3599	1.3332	1.2817	1.2567	1.2189	1.1951	1.1601	1.1360	1.1235	1.1013	1.0568	1.0375
100	1.3173	1.2996	1.2426	1.2259	1.1807	1.1658	1.1218	1.1078	1.0880	1.0732	1.0245	1.0109
150	1.2992	1.2865	1.2259	1.2131	1.1656	1.1532	1.1087	1.0959	1.0733	1.0622	1.0115	1.0004
200	1.2897	1.2785	1.2166	1.2054	1.1568	1.1462	1.1003	1.0896	1.0649	1.0551	1.0035	0.9944
250	1.2832	1.2734	1.2097	1.2002	1.1508	1.1415	1.0944	1.0851	1.0594	1.0511	0.9984	0.9904
300	1.2794	1.2696	1.2059	1.1970	1.1461	1.1378	1.0903	1.0816	1.0560	1.0478	0.9945	0.9871
350	1.2749	1.2665	1.2026	1.1943	1.1437	1.1355	1.0873	1.0794	1.0525	1.0451	0.9916	0.9852
400	1.2728	1.2636	1.1996	1.1917	1.1407	1.1333	1.0844	1.0774	1.0498	1.0437	0.9896	0.9835

**Table 4:** The adjusted values of *K*_*a*_ that guarantee *P*(*CARL*_*in*_ > *ARL*_0_ × (1 − *ϵ*)) = 1 − *α* of the synthetic $\bar{X}$ chart for different values of *ARL*_0_, *m* and *α* when *n* = 5 and *ϵ* = 10%.

	*α* = 0.05	*α* = 0.1	*α* = 0.05	*α* = 0.1	*α* = 0.05	*α* = 0.1	*α* = 0.05	*α* = 0.1	*α* = 0.05	*α* = 0.1	*α* = 0.05	*α* = 0.1
*m*	*ARL*_0_ = 200											
	(*δ*, *H*, *K*) = (0.2, 39, 1.1047)		(*δ*, *H*, *K*) = (0.4, 16, 1.0399)		(*δ*, *H*, *K*) = (0.6, 8, 0.9849)		(*δ*, *H*, *K*) = (0.8, 5, 0.9455)		(*δ*, *H*, *K*) = (1.0, 3, 0.9007)		(*δ*, *H*, *K*) = (1.4, 2, 0.8638)	
50	1.2179	1.1941	1.1457	1.1245	1.0861	1.0647	1.0428	1.0220	0.9932	0.9719	0.9524	0.9346
100	1.1792	1.1637	1.1102	1.0953	1.0519	1.0374	1.0088	0.9957	0.9625	0.9488	0.9221	0.9101
150	1.1638	1.1516	1.0955	1.0837	1.0377	1.0263	0.9962	0.9855	0.9487	0.9381	0.9099	0.9008
200	1.1549	1.1437	1.0874	1.0774	1.0299	1.0203	0.9882	0.9792	0.9411	0.9329	0.9031	0.8949
250	1.1491	1.1398	1.0814	1.0723	1.0245	1.0163	0.9833	0.9750	0.9374	0.9292	0.8985	0.8914
300	1.1449	1.1366	1.0778	1.0696	1.0206	1.0134	0.9800	0.9728	0.9336	0.9269	0.8947	0.8881
350	1.1416	1.1338	1.0743	1.0675	1.0175	1.0111	0.9776	0.9703	0.9308	0.9246	0.8930	0.8865
400	1.1391	1.1318	1.0723	1.0655	1.0158	1.0093	0.9752	0.9685	0.9290	0.9228	0.8908	0.8851
	*ARL*_0_ = 370.4											
	(*δ*, *H*, *K*) = (0.2, 56, 1.1831)		(*δ*, *H*, *K*) = (0.4, 22, 1.1176)		(*δ*, *H*, *K*) = (0.6, 10, 1.0576)		(*δ*, *H*, *K*) = (0.8, 6, 1.0166)		(*δ*, *H*, *K*) = (1.0, 4, 0.9829)		(*δ*, *H*, *K*) = (1.4, 2, 0.9226)	
50	1.3043	1.2786	1.2296	1.2073	1.1659	1.1425	1.1204	1.0985	1.0836	1.0621	1.0182	0.9973
100	1.2614	1.2463	1.1929	1.1765	1.1289	1.1133	1.0858	1.0705	1.0486	1.0360	0.9854	0.9724
150	1.2467	1.2325	1.1776	1.1644	1.1141	1.1022	1.0706	1.0594	1.0352	1.0245	0.9717	0.9613
200	1.2369	1.2256	1.1689	1.1579	1.1058	1.0956	1.0622	1.0533	1.0273	1.0181	0.9646	0.9557
250	1.2308	1.2209	1.1616	1.1530	1.1006	1.0908	1.0571	1.0485	1.0226	1.0142	0.9602	0.9514
300	1.2265	1.2172	1.1582	1.1494	1.0960	1.0875	1.0535	1.0462	1.0189	1.0108	0.9566	0.9487
350	1.2224	1.2142	1.1544	1.1473	1.0927	1.0855	1.0501	1.0434	1.0159	1.0083	0.9539	0.9471
400	1.2200	1.2122	1.1525	1.1449	1.0904	1.0837	1.0484	1.0413	1.0133	1.0070	0.9516	0.9454
	*ARL*_0_ = 500											
	(*δ*, *H*, *K*) = (0.2, 68, 1.2209)		(*δ*, *H*, *K*) = (0.4, 25, 1.1519)		(*δ*, *H*, *K*) = (0.6, 11, 1.0906)		(*δ*, *H*, *K*) = (0.8, 6, 1.0427)		(*δ*, *H*, *K*) = (1.0, 4, 1.0094)		(*δ*, *H*, *K*) = (1.4, 2, 0.9502)	
50	1.3463	1.3191	1.2689	1.2454	1.2015	1.1793	1.1505	1.1269	1.1127	1.0909	1.0475	1.0274
100	1.3037	1.2861	1.2293	1.2132	1.1634	1.1483	1.1132	1.0982	1.0772	1.0633	1.0147	1.0006
150	1.2862	1.2718	1.2133	1.2009	1.1490	1.1365	1.0981	1.0869	1.0634	1.0521	1.0012	0.9901
200	1.2764	1.2649	1.2047	1.1935	1.1405	1.1299	1.0903	1.0799	1.0553	1.0457	0.9932	0.9844
250	1.2703	1.2596	1.1984	1.1881	1.1346	1.1249	1.0854	1.0759	1.0501	1.0417	0.9892	0.9805
300	1.2652	1.2561	1.1936	1.1849	1.1305	1.1218	1.0805	1.0726	1.0461	1.0381	0.9849	0.9774
350	1.2625	1.2531	1.1905	1.1822	1.1264	1.1196	1.0770	1.0701	1.0433	1.0359	0.9820	0.9755
400	1.2593	1.2501	1.1879	1.1798	1.1247	1.1176	1.0753	1.0682	1.0403	1.0340	0.9800	0.9733

**Table 5:** The adjusted values of *K*_*a*_ that guarantee *P*(*CARL*_*in*_ > *ARL*_0_ × (1 − *ϵ*)) = 1 − *α* of the synthetic $\bar{X}$ chart for different values of *ARL*_0_, *m* and *α* when *n* = 5 and *ϵ* = 20%.

	*α* = 0.05	*α* = 0.1	*α* = 0.05	*α* = 0.1	*α* = 0.05	*α* = 0.1	*α* = 0.05	*α* = 0.1	*α* = 0.05	*α* = 0.1	*α* = 0.05	*α* = 0.1
*m*	*ARL*_0_ = 200											
	(*δ*, *H*, *K*) = (0.2, 36, 1.0884)		(*δ*, *H*, *K*) = (0.4, 15, 1.0242)		(*δ*, *H*, *K*) = (0.6, 8, 0.9740)		(*δ*, *H*, *K*) = (0.8, 5, 0.9344)		(*δ*, *H*, *K*) = (1.0, 3, 0.8894)		(*δ*, *H*, *K*) = (1.4, 2, 0.8522)	
50	1.1989	1.1767	1.1287	1.1064	1.0736	1.0530	1.0299	1.0104	0.9819	0.9615	0.9394	0.9222
100	1.1624	1.1465	1.0938	1.0790	1.0399	1.0260	0.9984	0.9836	0.9498	0.9368	0.9097	0.8985
150	1.1465	1.1345	1.0799	1.0678	1.0264	1.0151	0.9848	0.9739	0.9368	0.9273	0.8973	0.8879
200	1.1382	1.1273	1.0709	1.0613	1.0190	1.0090	0.9766	0.9681	0.9299	0.9216	0.8912	0.8826
250	1.1318	1.1233	1.0657	1.0563	1.0132	1.0047	0.9719	0.9644	0.9254	0.9176	0.8868	0.8792
300	1.1282	1.1193	1.0612	1.0537	1.0094	1.0018	0.9687	0.9610	0.9220	0.9151	0.8833	0.8765
350	1.1248	1.1168	1.0589	1.0511	1.0066	0.9996	0.9653	0.9591	0.9196	0.9129	0.8807	0.8745
400	1.1222	1.1149	1.0559	1.0489	1.0040	0.9980	0.9637	0.9575	0.9175	0.9112	0.8788	0.8733
	*ARL*_0_ = 370.4											
	(*δ*, *H*, *K*) = (0.2, 52, 1.1682)		(*δ*, *H*, *K*) = (0.4, 20, 1.1006)		(*δ*, *H*, *K*) = (0.6, 10, 1.0474)		(*δ*, *H*, *K*) = (0.8, 6, 1.0062)		(*δ*, *H*, *K*) = (1.0, 4, 0.9723)		(*δ*, *H*, *K*) = (1.4, 2, 0.9116)	
50	1.2883	1.2625	1.2131	1.1895	1.1539	1.1324	1.1089	1.0883	1.0727	1.0511	1.0038	0.9852
100	1.2472	1.2303	1.1753	1.1590	1.1185	1.1026	1.0748	1.0603	1.0376	1.0246	0.9731	0.9599
150	1.2309	1.2175	1.1587	1.1476	1.1035	1.0915	1.0595	1.0486	1.0244	1.0137	0.9605	0.9504
200	1.2218	1.2101	1.1505	1.1404	1.0945	1.0850	1.0517	1.0424	1.0160	1.0070	0.9533	0.9447
250	1.2156	1.2052	1.1451	1.1359	1.0896	1.0808	1.0465	1.0384	1.0118	1.0032	0.9485	0.9405
300	1.2098	1.2013	1.1409	1.1320	1.0854	1.0776	1.0428	1.0351	1.0075	1.0006	0.9451	0.9377
350	1.2078	1.1988	1.1377	1.1292	1.0825	1.0744	1.0400	1.0326	1.0046	0.9975	0.9422	0.9361
400	1.2048	1.1968	1.1347	1.1275	1.0794	1.0731	1.0377	1.0307	1.0026	0.9960	0.9401	0.9338
	*ARL*_0_ = 500											
	(*δ*, *H*, *K*) = (0.2, 63, 1.2062)		(*δ*, *H*, *K*) = (0.4, 24, 1.1393)		(*δ*, *H*, *K*) = (0.6, 11, 1.0807)		(*δ*, *H*, *K*) = (0.8, 6, 1.0325)		(*δ*, *H*, *K*) = (1.0, 4, 0.9991)		(*δ*, *H*, *K*) = (1.4, 2, 0.9394)	
50	1.3289	1.3038	1.2555	1.2317	1.1894	1.1683	1.1394	1.1155	1.1015	1.0792	1.0358	1.0154
100	1.2872	1.2704	1.2157	1.2002	1.1537	1.1382	1.1029	1.0881	1.0662	1.0522	1.0024	0.9895
150	1.2710	1.2566	1.2007	1.1873	1.1387	1.1266	1.0882	1.0762	1.0525	1.0411	0.9898	0.9787
200	1.2605	1.2491	1.1913	1.1800	1.1303	1.1190	1.0792	1.0697	1.0448	1.0349	0.9821	0.9730
250	1.2541	1.2445	1.1853	1.1754	1.1243	1.1153	1.0746	1.0652	1.0394	1.0310	0.9773	0.9689
300	1.2503	1.2406	1.1811	1.1723	1.1204	1.1118	1.0704	1.0624	1.0351	1.0281	0.9736	0.9668
350	1.2463	1.2381	1.1777	1.1696	1.1172	1.1092	1.0669	1.0596	1.0321	1.0254	0.9713	0.9640
400	1.2442	1.2361	1.1742	1.1671	1.1148	1.1068	1.0649	1.0581	1.0304	1.0233	0.9690	0.9626

By using these adjusted control limits, the conditional in-control performance of the synthetic $\bar{X}$ chart can be improved greatly. While at the same time, the out-of-control performance of the synthetic $\bar{X}$ chart deteriorates to some extent. This fact has been noted in some related researches as the price to pay for the improvement in the conditional in-control performance (see Saleh et al. [13] and Hu et al. [30]). However, by increasing the values of *ϵ* or *α* (or both) in Eq (9), we can improve the conditional out-of-control performance of the synthetic $\bar{X}$ chart, but at the same time, deteriorating the conditional in-control performances to some extent. In the next section, the conditional in- and out-of-control performances of the synthetic chart using the unadjusted and adjusted control limits are investigated.

## 5 Comparisons of the adjusted and unadjusted synthetic $\bar{X}$ charts

In this section, a comprehensive evaluation of the synthetic $\bar{X}$ chart for different shift sizes, using the unadjusted and adjusted control limits are conducted. Moreover, our results are also compared with the conditional performances of the synthetic $\bar{X}$ chart using the bootstrap approach in Hu et al. [30].

When *ARL*_0_ = 200, *α* = 0.1 and *n* = 5, for different values of *m* ∈ {50, 100, 150, 200, 250, 300} and *δ* ∈ {0.2, 0.4, 0.6, 0.8, 1.0, 1.4}, Table 6 presents several percentiles of the conditional in- and out-of-control *ARL* of the synthetic chart in the bracket in each entry. All the bolded values in the table are the 10% percentiles of the *CARL*_*in*_ (*CARL*_*in*,10%_) that can be attained with a high probability 90%, using the adjusted parameters. The adjusted parameters of the synthetic chart can be obtained from the upper part in Table 3, where the adjusted values *K*_*a*_ are presented when *α* = 0.1, *ARL*_0_ = 200 and *m* ∈ {50, 100, 150, 200, 250, 300, 350, 400}. For example, when *m* = 50 and *δ* = 0.4, the optimized values (*H*, *K*) of the synthetic $\bar{X}$ chart in the known process parameters case are (17,1.0540) (unadjusted parameter). The *CARL*_*in*,10%_ and the 10% percentiles of the out-of-control *CARL* (*CARL*_*out*,10%_) values are 84.81 and 10.26 (see row 5, column 4 in Table 6), respectively, using the unadjusted limits. To guarantee that the *CARL*_*in*_ values exceed the desired *ARL*_0_ = 200, the adjusted control limits (*H*, *K*_*a*_) are suggested as (17,1.1384) (see column 5, row 4 in Table 3). Then we can note that *CARL*_*in*,10%_ and *CARL*_*out*,10%_ are 201.19 and 16.41 (see row 5, column 5 in Table 6), respectively. As expected, the value *CARL*_*in*,10%_ = 201.19 is close to the desired *ARL*_0_ which we hope the *CARL*_*in*_ values of the synthetic chart exceeds with a probability 90%. From Table 6, several conclusions can be made as follows:

- All the *CARL*_*in*,10%_ values using the adjusted parameters are close to the desired *ARL*_0_. For example, when *m* = 50, the *CARL*_*in*,10%_ values are {200.32,201.19,200.22,201.19,200.28,200.74} (see the bolded values in row 5) for different values of (*δ*, *H*, *K*). Moreover, all the percentiles of the *CARL*_*in*_ values using the adjusted parameters are larger than the corresponding ones using the unadjusted parameters. For example, when *m* = 50, the 25% and 50% percentiles of the *CARL*_*in*_ values using the adjusted limits are 288.96 and 462.05 (see column 5), respectively, which are larger than the corresponding values 119.28 and 175.71 (see column 4) using the unadjusted limits.
- When the process shift size is small, the difference between the percentiles of the *CARL*_*out*_ values corresponding to the adjusted and unadjusted limits is large. For example, when *δ* = 0.2, the 10% and 50% percentiles of the *CARL*_*out*_ values using the adjusted limits are 67.70 and 178.79, respectively, and the 10% and 50% percentiles of the *CARL*_*out*_ values using the unadjusted limits are 34.45 and 74.19, respectively. This is the result of improving the *CARL*_*in*_ performance using the adjusted control limits. With the increase in the shift size *δ*, this difference becomes small. For moderate to large shift sizes in the process, the percentiles of the *CARL*_*out*_ performance of the synthetic $\bar{X}$ chart using the adjusted and unadjusted limits are comparable. For example, when *δ* = 0.8 (see columns 8 and 9), the 10% and 50% percentiles of the *CARL*_*out*_ values using the adjusted limits are 2.83 and 3.95, respectively, and the 10% and 50% percentiles of the *CARL*_*out*_ values using the unadjusted limits are 2.32 and 3.05, respectively. The difference is generally acceptable in practice. However, we may note that, by using the adjusted limits, the *CARL*_*in*_ performances of the chart improve substantially compared with the ones using the unadjusted limits. This fact shows that, for moderate to large shift sizes, the adjusted limits can get a good *CARL*_*in*_ and *CARL*_*out*_ performances of the synthetic $\bar{X}$ chart.
- With the increase in the available number *m* of Phase I samples, the difference between the percentiles of the *CARL*_*in*_ (or *CARL*_*out*_) corresponding the adjusted and unadjusted limits becomes small. For example, when *δ* = 0.4 and *m* = 50, the 50% percentiles of the *CARL*_*in*_ and *CARL*_*out*_ values for the unadjusted limits and adjusted limits cases are (175.71, 18.83) and (462.05, 33.84), respectively. While if *m* = 300, the 50% percentiles of the *CARL*_*in*_ and *CARL*_*out*_ values for the unadjusted limits and adjusted limits cases are (195.97, 19.11) and (275.65, 23.38), respectively (see columns 4 and 5).

**Table 6:** Some percentiles of the in- and out-of-control *CARL* values of the adjusted and unadjusted synthetic $\bar{X}$ chart when *ARL*_0_ = 200, *n* = 5 and *α* = 0.1.

	(*δ*, *H*, *K*) = (0.2, 41, 1.1177)		(*δ*, *H*, *K*) = (0.4, 17, 1.0540)		(*δ*, *H*, *K*) = (0.6, 8, 0.9945)		(*δ*, *H*, *K*) = (0.8, 5, 0.9553)		(*δ*, *H*, *K*) = (1.0, 3, 0.9108)		(*δ*, *H*, *K*) = (1.4, 2, 0.8741)	
	Unadjusted	Adjusted	Unadjusted	Adjusted	Unadjusted	Adjusted	Unadjusted	Adjusted	Unadjusted	Adjusted	Unadjusted	Adjusted
Percentiles	*m* = 50											
5%	(68.31, 28.24)	(155.35, 53.07)	(69.77, 8.94)	(161.68, 13.76)	(74.10, 3.85)	(162.09, 5.09)	(79.45, 2.17)	(164.11, 2.62)	(82.48, 1.49)	(164.08, 1.67)	(89.97, 1.08)	(166.49, 1.12)
10%	(82.59, 34.45)	(**200.32**, 67.70)	(84.81, 10.26)	(**201.19**, 16.41)	(89.62, 4.26)	(**200.22**, 5.78)	(94.89, 2.32)	(**201.19**, 2.83)	(98.22, 1.56)	(**200.28**, 1.77)	(105.29, 1.10)	(**200.74**, 1.13)
15%	(94.14, 39.59)	(234.71, 80.76)	(97.45, 11.45)	(231.33, 18.63)	(101.84, 4.58)	(230.11, 6.28)	(106.47, 2.43)	(228.15, 3.00)	(109.83, 1.60)	(225.41, 1.83)	(116.49, 1.11)	(226.55, 1.14)
20%	(105.68, 44.26)	(269.98, 93.52)	(108.47, 12.49)	(259.46, 20.63)	(112.73, 4.86)	(258.74, 6.78)	(117.02, 2.52)	(256.01, 3.14)	(120.48, 1.65)	(250.62, 1.88)	(126.28, 1.11)	(248.79, 1.16)
25%	(116.99, 48.95)	(303.39, 106.61)	(119.28, 13.46)	(288.96, 22.60)	(122.79, 5.11)	(286.93, 7.24)	(126.76, 2.61)	(283.31, 3.28)	(130.70, 1.69)	(275.69, 1.94)	(135.39, 1.12)	(271.32, 1.16)
50%	(176.06, 74.19)	(505.56, 178.79)	(175.71, 18.83)	(462.05, 33.84)	(177.46, 6.47)	(440.29, 9.65)	(179.75, 3.05)	(417.16, 3.95)	(180.79, 1.86)	(400.63, 2.19)	(183.26, 1.15)	(381.56, 1.21)
75%	(270.01, 116.48)	(852.47, 315.38)	(263.42, 27.09)	(743.84, 52.87)	(258.69, 8.45)	(680.00, 13.21)	(257.32, 3.64)	(624.97, 4.91)	(252.81, 2.09)	(584.19, 2.54)	(249.26, 1.19)	(547.78, 1.27)
90%	(403.94, 182.44)	(1403.91, 553.67)	(397.90, 39.66)	(1172.15, 83.36)	(364.61, 11.02)	(1029.79, 18.30)	(359.77, 4.37)	(921.56, 6.10)	(344.45, 2.35)	(840.45, 2.94)	(331.71, 1.24)	(770.40, 1.33)
95%	(519.39, 243.72)	(1892.37, 792.47)	(507.72, 50.50)	(1577.19, 111.27)	(457.70, 13.09)	(1359.82, 22.76)	(449.19, 4.87)	(1156.03, 7.01)	(421.23, 2.54)	(1043.27, 3.20)	(394.04, 1.27)	(942.49, 1.38)
	*m* = 100											
5%	(93.98, 36.53)	(167.93, 57.54)	(96.71, 10.98)	(172.93, 14.96)	(101.49, 4.39)	(172.26, 5.42)	(106.24, 2.38)	(173.66, 2.71)	(109.46, 1.59)	(177.64, 1.72)	(113.20, 1.10)	(176.82, 1.12)
10%	(108.23, 42.57)	(**200.10**, 68.97)	(112.07, 12.27)	(**201.20**, 17.10)	(116.25, 4.76)	(**200.32**, 5.91)	(119.80, 2.50)	(**200.59**, 2.87)	(123.69, 1.64)	(**200.80**, 1.79)	(127.68, 1.11)	(**200.81**, 1.14)
15%	(119.10, 47.67)	(223.54, 78.19)	(123.98, 13.37)	(223.98, 18.55)	(126.85, 5.03)	(220.79, 6.30)	(130.75, 2.59)	(220.07, 2.97)	(134.16, 1.68)	(218.63, 1.84)	(136.81, 1.12)	(217.78, 1.14)
20%	(129.22, 51.77)	(246.20, 86.14)	(133.34, 14.25)	(245.35, 19.98)	(135.94, 5.26)	(239.46, 6.64)	(139.64, 2.67)	(236.83, 3.08)	(143.26, 1.71)	(233.69, 1.88)	(145.25, 1.12)	(231.40, 1.15)
25%	(138.63, 56.09)	(266.10, 93.89)	(142.06, 15.10)	(264.88, 21.31)	(145.17, 5.46)	(257.79, 6.94)	(148.52, 2.74)	(252.71, 3.18)	(151.60, 1.74)	(247.65, 1.91)	(152.84, 1.13)	(245.25, 1.16)
50%	(187.22, 76.75)	(370.85, 135.09)	(187.35, 19.22)	(361.25, 28.17)	(188.55, 6.50)	(339.30, 8.41)	(189.60, 3.06)	(330.02, 3.61)	(190.66, 1.86)	(321.15, 2.07)	(188.84, 1.15)	(308.30, 1.19)
75%	(254.29, 107.03)	(521.28, 198.19)	(250.29, 24.82)	(496.04, 37.90)	(245.40, 7.79)	(452.45, 10.41)	(242.06, 3.46)	(437.29, 4.15)	(239.29, 2.02)	(414.19, 2.27)	(234.55, 1.18)	(392.03, 1.23)
90%	(336.54, 145.94)	(716.56, 283.35)	(327.53, 31.84)	(669.68, 50.04)	(316.77, 9.37)	(591.36, 12.72)	(307.95, 3.90)	(561.24, 4.78)	(295.80, 2.18)	(521.89, 2.48)	(285.90, 1.21)	(489.04, 1.26)
95%	(397.02, 177.77)	(870.30, 352.94)	(391.06, 37.52)	(804.67, 59.77)	(367.69, 10.52)	(705.57, 14.51)	(351.41, 4.20)	(648.80, 5.22)	(339.16, 2.29)	(603.90, 2.64)	(324.79, 1.23)	(565.05, 1.29)
	*m* = 150											
5%	(108.13, 41.60)	(175.36, 60.53)	(111.28, 12.13)	(176.26, 15.39)	(116.56, 4.76)	(179.46, 5.56)	(118.48, 2.47)	(179.31, 2.76)	(123.87, 1.63)	(178.72, 1.74)	(127.40, 1.11)	(181.96, 1.13)
10%	(122.32, 47.31)	(**200.06**, 70.10)	(124.52, 13.28)	(**200.78**, 17.11)	(129.60, 5.06)	(**200.31**, 6.01)	(131.56, 2.58)	(**200.22**, 2.89)	(135.97, 1.67)	(**200.17**, 1.79)	(139.53, 1.12)	(**200.72**, 1.14)
15%	(133.23, 51.67)	(218.73, 77.51)	(135.64, 14.20)	(220.04, 18.41)	(139.47, 5.30)	(216.75, 6.34)	(141.54, 2.66)	(215.97, 2.99)	(145.05, 1.71)	(214.34, 1.84)	(148.43, 1.12)	(214.64, 1.15)
20%	(142.53, 55.64)	(236.62, 83.35)	(145.48, 14.97)	(235.38, 19.56)	(148.09, 5.50)	(231.30, 6.61)	(149.58, 2.73)	(230.15, 3.07)	(153.30, 1.73)	(226.58, 1.87)	(156.16, 1.13)	(225.72, 1.15)
25%	(150.79, 59.14)	(250.86, 89.32)	(153.40, 15.66)	(249.55, 20.70)	(156.09, 5.67)	(244.39, 6.85)	(157.39, 2.79)	(243.08, 3.15)	(160.36, 1.76)	(238.69, 1.90)	(162.96, 1.13)	(236.51, 1.16)
50%	(190.47, 76.59)	(328.66, 119.58)	(191.79, 19.10)	(319.78, 25.86)	(191.83, 6.51)	(307.52, 7.97)	(191.72, 3.05)	(301.21, 3.50)	(192.66, 1.86)	(290.73, 2.03)	(193.45, 1.15)	(285.12, 1.18)
75%	(244.33, 101.01)	(433.02, 161.80)	(242.70, 23.64)	(413.70, 32.85)	(239.09, 7.53)	(387.34, 9.36)	(234.53, 3.37)	(376.22, 3.90)	(232.01, 1.99)	(354.79, 2.18)	(230.84, 1.18)	(345.68, 1.21)
90%	(310.46, 129.75)	(559.72, 215.16)	(300.30, 29.04)	(526.52, 41.01)	(288.68, 8.64)	(477.48, 10.93)	(282.89, 3.71)	(457.13, 4.35)	(275.70, 2.12)	(426.76, 2.33)	(270.11, 1.20)	(416.20, 1.24)
95%	(358.90, 151.07)	(654.00, 257.64)	(341.65, 33.24)	(609.68, 47.11)	(326.03, 9.44)	(542.55, 12.08)	(320.85, 3.94)	(517.14, 4.65)	(307.48, 2.20)	(478.69, 2.43)	(299.64, 1.21)	(460.75, 1.25)
	*m* = 200											
5%	(119.19, 45.63)	(177.67, 62.73)	(121.11, 12.75)	(180.64, 15.89)	(124.37, 4.92)	(182.34, 5.67)	(128.68, 2.55)	(182.77, 2.80)	(131.80, 1.66)	(184.06, 1.76)	(136.15, 1.11)	(183.12, 1.13)
10%	(132.02, 50.81)	(**200.01**, 70.67)	(134.73, 13.85)	(**200.03**, 17.38)	(137.07, 5.21)	(**200.33**, 6.05)	(140.69, 2.65)	(**200.83**, 2.91)	(143.93, 1.70)	(**201.03**, 1.80)	(146.52, 1.12)	(**200.09**, 1.14)
15%	(141.57, 54.70)	(216.70, 77.03)	(144.11, 14.75)	(215.16, 18.55)	(146.25, 5.43)	(214.91, 6.31)	(149.23, 2.71)	(214.20, 3.00)	(152.32, 1.73)	(213.48, 1.84)	(154.46, 1.13)	(211.21, 1.15)
20%	(149.35, 58.31)	(229.64, 82.40)	(152.42, 15.46)	(227.92, 19.56)	(154.39, 5.60)	(226.70, 6.54)	(157.04, 2.77)	(225.52, 3.07)	(159.06, 1.75)	(223.54, 1.87)	(160.99, 1.13)	(220.62, 1.15)
25%	(156.90, 61.68)	(241.62, 87.15)	(160.10, 16.09)	(239.82, 20.48)	(161.75, 5.77)	(238.12, 6.76)	(163.62, 2.83)	(236.45, 3.14)	(165.61, 1.77)	(233.30, 1.89)	(167.29, 1.13)	(229.84, 1.16)
50%	(193.80, 77.07)	(304.16, 111.68)	(194.10, 19.10)	(299.21, 24.81)	(193.96, 6.51)	(288.35, 7.69)	(194.37, 3.06)	(283.00, 3.42)	(195.08, 1.86)	(275.92, 2.00)	(195.02, 1.15)	(269.21, 1.18)
75%	(239.45, 97.68)	(386.75, 143.88)	(237.43, 22.97)	(372.87, 30.09)	(232.96, 7.37)	(349.78, 8.87)	(231.94, 3.33)	(340.74, 3.76)	(229.45, 1.97)	(329.19, 2.12)	(226.99, 1.17)	(318.02, 1.20)
90%	(291.75, 120.68)	(481.47, 183.04)	(286.70, 27.64)	(454.06, 36.32)	(276.60, 8.30)	(424.29, 10.18)	(271.58, 3.61)	(407.28, 4.11)	(266.59, 2.07)	(383.26, 2.25)	(259.26, 1.19)	(370.47, 1.22)
95%	(324.89, 137.86)	(552.15, 214.36)	(319.72, 30.96)	(509.74, 40.99)	(307.93, 8.95)	(474.85, 10.99)	(299.74, 3.80)	(451.71, 4.35)	(292.80, 2.15)	(421.42, 2.33)	(283.38, 1.20)	(406.11, 1.24)
	*m* = 250											
5%	(124.56, 47.59)	(181.44, 64.01)	(128.95, 13.31)	(181.21, 16.15)	(131.42, 5.04)	(183.03, 5.78)	(133.10, 2.59)	(183.34, 2.81)	(138.16, 1.68)	(185.07, 1.77)	(141.70, 1.12)	(186.30, 1.13)
10%	(137.05, 52.89)	(**200.99**, 71.45)	(139.64, 14.42)	(**200.50**, 17.48)	(143.63, 5.31)	(**201.04**, 6.10)	(145.21, 2.69)	(**200.20**, 2.91)	(148.70, 1.71)	(**200.19**, 1.81)	(151.96, 1.12)	(**200.25**, 1.14)
15%	(145.94, 56.44)	(213.03, 76.45)	(148.63, 15.22)	(213.75, 18.50)	(152.37, 5.52)	(212.87, 6.33)	(154.06, 2.75)	(211.50, 2.99)	(156.53, 1.74)	(211.04, 1.84)	(159.44, 1.13)	(210.34, 1.15)
20%	(153.75, 59.59)	(225.14, 81.36)	(156.11, 15.84)	(225.48, 19.36)	(159.80, 5.68)	(223.67, 6.53)	(160.89, 2.80)	(221.39, 3.06)	(163.42, 1.76)	(220.33, 1.87)	(165.87, 1.13)	(218.77, 1.15)
25%	(160.82, 62.65)	(235.46, 85.51)	(163.47, 16.45)	(235.67, 20.16)	(166.07, 5.82)	(232.99, 6.71)	(167.65, 2.85)	(230.43, 3.12)	(169.63, 1.78)	(228.60, 1.89)	(171.58, 1.14)	(227.15, 1.16)
50%	(194.25, 76.93)	(287.86, 106.24)	(194.07, 19.10)	(284.37, 23.82)	(195.05, 6.49)	(277.28, 7.55)	(196.16, 3.06)	(271.75, 3.37)	(195.81, 1.87)	(266.43, 1.99)	(196.41, 1.15)	(262.33, 1.17)
75%	(234.67, 94.43)	(352.10, 133.71)	(232.67, 22.43)	(344.65, 28.39)	(231.06, 7.28)	(331.65, 8.56)	(229.38, 3.30)	(322.07, 3.67)	(226.19, 1.96)	(311.42, 2.09)	(226.03, 1.17)	(301.97, 1.19)
90%	(278.86, 114.42)	(425.86, 164.24)	(274.58, 26.05)	(410.94, 33.50)	(268.50, 8.14)	(391.96, 9.62)	(264.22, 3.55)	(373.43, 3.97)	(257.70, 2.05)	(358.79, 2.20)	(255.76, 1.19)	(344.22, 1.21)
95%	(310.97, 128.52)	(479.23, 185.23)	(305.13, 28.51)	(454.74, 37.19)	(295.45, 8.65)	(431.09, 10.28)	(289.40, 3.71)	(407.67, 4.17)	(277.86, 2.11)	(388.15, 2.28)	(275.99, 1.20)	(373.45, 1.23)
	*m* = 300											
5%	(130.95, 50.53)	(181.79, 64.94)	(132.04, 13.55)	(183.69, 16.41)	(136.11, 5.16)	(183.78, 5.78)	(139.65, 2.63)	(185.73, 2.82)	(143.35, 1.69)	(186.23, 1.33)	(147.21, 1.12)	(185.87, 1.13)
10%	(142.07, 55.10)	(**200.11**, 71.96)	(143.55, 14.65)	(**200.99**, 17.67)	(147.59, 5.41)	(**200.31**, 6.11)	(150.43, 2.71)	(**201.26**, 2.93)	(153.82, 1.73)	(**200.18**, 1.35)	(156.18, 1.13)	(**200.27**, 1.14)
15%	(151.03, 58.81)	(213.15, 76.94)	(152.00, 15.40)	(212.80, 18.55)	(154.88, 5.60)	(211.56, 6.33)	(157.92, 2.77)	(211.13, 3.00)	(161.35, 1.75)	(210.43, 1.36)	(163.17, 1.13)	(210.11, 1.15)
20%	(158.07, 61.73)	(223.70, 81.37)	(159.77, 16.05)	(224.09, 19.36)	(161.23, 5.75)	(221.58, 6.52)	(164.57, 2.82)	(219.88, 3.06)	(167.49, 1.77)	(219.08, 1.37)	(169.46, 1.13)	(217.86, 1.15)
25%	(164.72, 64.49)	(233.86, 85.23)	(166.07, 16.63)	(233.02, 20.05)	(167.59, 5.89)	(229.78, 6.69)	(170.64, 2.87)	(227.85, 3.11)	(172.75, 1.79)	(226.61, 1.38)	(174.60, 1.14)	(224.35, 1.15)
50%	(195.61, 77.39)	(282.00, 103.61)	(195.97, 19.11)	(275.65, 23.38)	(195.23, 6.50)	(269.70, 7.45)	(196.73, 3.06)	(263.80, 3.33)	(196.60, 1.87)	(260.13, 1.42)	(197.01, 1.15)	(254.07, 1.17)
75%	(232.62, 93.37)	(340.48, 128.09)	(231.24, 22.22)	(328.19, 27.38)	(227.89, 7.22)	(317.16, 8.32)	(226.31, 3.28)	(305.63, 3.59)	(225.26, 1.95)	(300.08, 1.46)	(223.27, 1.17)	(291.09, 1.19)
90%	(274.91, 111.26)	(405.85, 155.28)	(268.15, 25.50)	(383.69, 31.72)	(261.90, 7.99)	(368.15, 9.28)	(258.25, 3.51)	(350.53, 3.86)	(254.81, 2.03)	(342.13, 1.49)	(249.50, 1.18)	(328.39, 1.21)
95%	(301.89, 123.12)	(447.42, 174.80)	(293.66, 27.80)	(425.25, 34.86)	(283.56, 8.50)	(404.37, 9.86)	(280.78, 3.65)	(381.77, 4.03)	(274.43, 2.09)	(369.10, 1.52)	(267.25, 1.19)	(351.99, 1.22)

**Fig 1:**
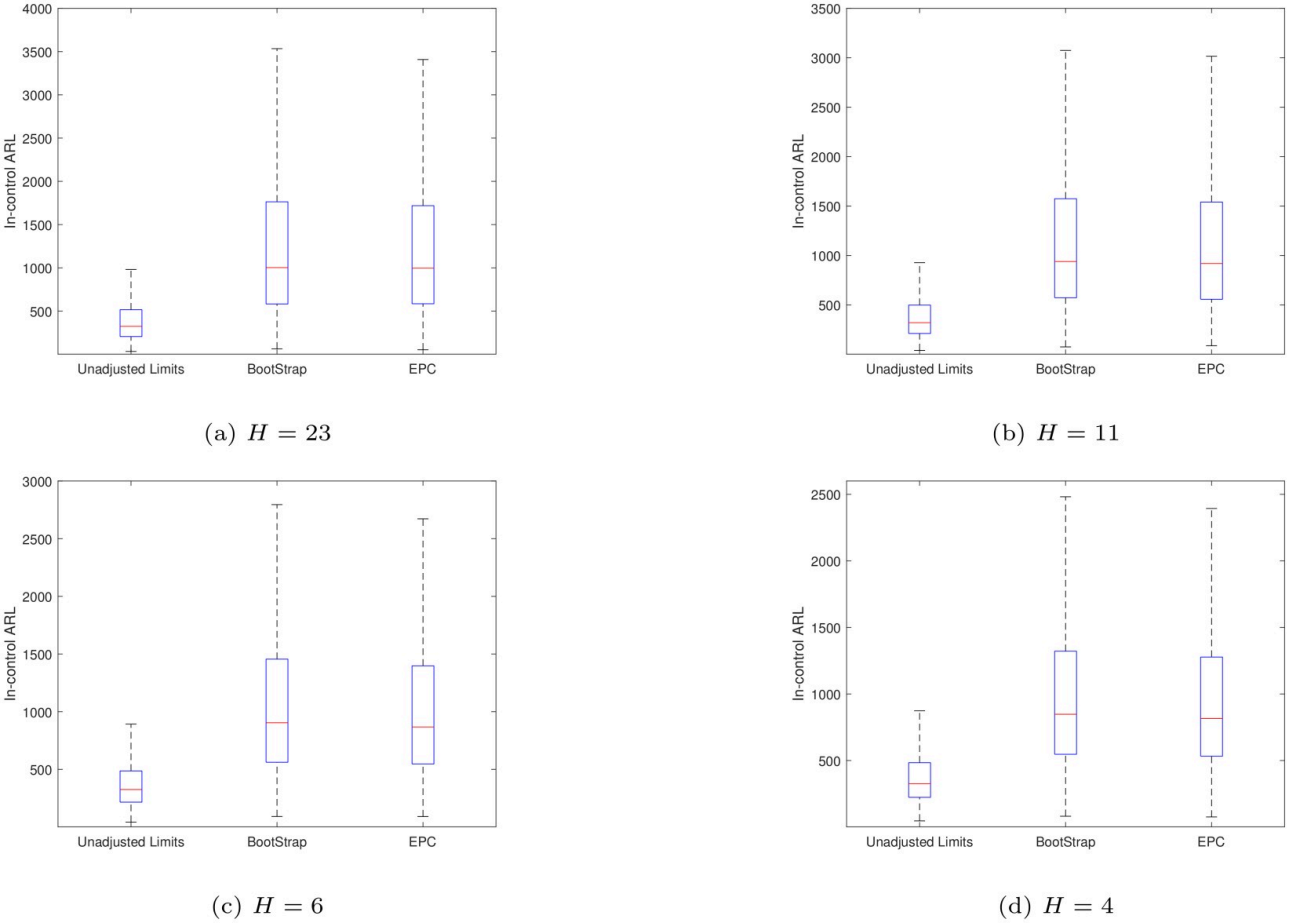
The boxplots of the *CARL*_*in*_ values of the synthetic $\bar{X}$ chart for different values of *H* when *ARL*_0_ = 370.4, *m* = 50 and *n* = 5.

**Fig 2:**
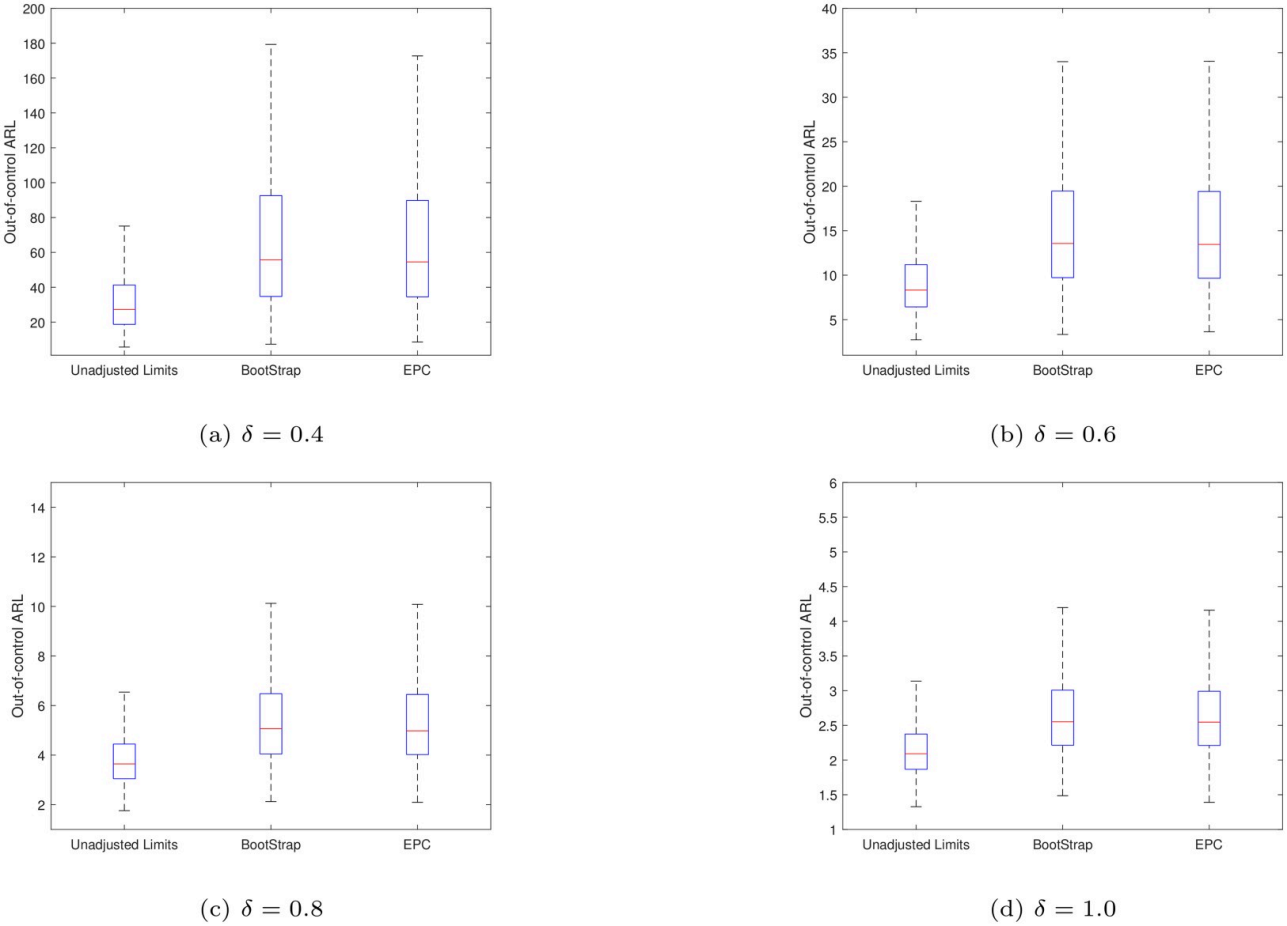
The boxplots of the *CARL*_*out*_ values of the synthetic $\bar{X}$ chart different values of *δ* when *ARL*_0_ = 370.4, *m* = 50 and *n* = 5.

To validate the method in this paper, a comparison is made with the results in Hu et al. [30]. Figs 1 and 2 present the boxplots of the conditional in- and out-of-control *ARL* values of the synthetic $\bar{X}$ chart with the unadjusted and adjusted limits when *ARL*_0_ = 370.4, *m* = 50, *n* = 5 and *α* = 0.1. It is noted that the unadjusted limits (*H*, *K*) studied here are optimized for different shift size *δ* ∈ {0.4, 0.6, 0.8, 1.0} when the process parameters are *known*, leading to the following values (*H*, *K*)∈{(23, 1.1297), (11, 1.0741), (6, 1.0259), (4, 0.9923)}, respectively. If the control limits of the synthetic chart are adjusted using the bootstrap approach in Hu et al. [30], they are denoted as “BootStrap”. Otherwise, if the control limits are adjusted using the *EPC* approach in this paper, it is denoted as “EPC”.

From Fig 1, as expected, both the “BootStrap” and “EPC” adjusted limits guarantee about 90% of the synthetic $\bar{X}$ chart to have *CARL*_*in*_ values larger than *ARL*_0_ = 370.4 and both approaches have comparable *CARL*_*in*_ performances. It can also be seen that most of the *CARL*_*in*_ values of the synthetic $\bar{X}$ chart with adjusted limits are larger than the ones with unadjusted limits. When the process is out-of-control, from Fig 2, it can be noted that the *CARL*_*out*_ of the synthetic chart using the “BootStrap” and “EPC” approaches performs similarly. When the process mean shift size increases (*δ* ≥ 0.6), using the adjusted limits only causes a small increase in the *CARL*_*out*_ values compared with the ones of the unadjusted limits cases. So it can be concluded that the *EPC* approach performs similarly with the bootstrap approach and the former also has the advantage of less computational effort than the bootstrap approach.

It is noted that the aim of the adjusted control limits is to guarantee a minimum in-control performance with a specified probability. However, the resulting out-of-control performance should not be ignored, as detecting out-of-control situations is still the main purpose when using control charts. So, the user should compromise between the risk of a large false-alarm rate and a poor out-of-control performance. Actually, if the in-control performance of the synthetic $\bar{X}$ chart is not deemed sufficiently enough, two alternative options are possible. The first one is to increase the amount of Phase I data *m* or the sample size *n* (or both) and the second one is to be more lenient on the guaranteed in-control performance by adjusting the control limits of the chart. Since the amount of Phase I samples or samples size are first fixed in practice, the second option has to be adopted. That is, one chooses to be more lenient on the adjustment of control limits. Then the adjustment of control limits are the same when they are obtained using different approaches and the resulting performances of the synthetic chart using the EPC and the bootstrap approaches are exactly the same.

## 6 Conclusion

In this paper, the conditional *ARL* performance of the synthetic $\bar{X}$ chart is studied using the *EPC*. Some recommendations are made on the number *m* of Phase I samples to guarantee the *CARL*_*in*_ values larger than the desired *ARL*_0_ with a specified high probability. Using the *EPC*, it is noted that the minimum number *m* of Phase I samples needed to guarantee the *CARL*_*in*_ performance is larger than practically available. Then, instead of using the bootstrap approach, we adjust the control limits by approximating the empirical distribution of the *CARL*_*in*_ values. The results show that the method used in this paper can get similar performance as the bootstrap approach and moreover, it is computationally faster than the bootstrap approach. Adjusting the control limits of the synthetic chart can improve the *CARL*_*in*_ performances, while at the same time, deteriorating the *CARL*_*out*_ performance. Our results show that for moderate to large shift sizes (*δ* ≥ 0.6), the difference between the *CARL*_*out*_ performance of the adjusted and unadjusted limits cases is small. Moreover, by choosing the values of *ϵ* or *α* (or both) in Eq (9) and using the *EPC* approach to get the adjusted control limits, practitioners can balance the *CARL*_*in*_ and *CARL*_*out*_ performances of the synthetic $\bar{X}$ chart.

All the results presented above is based on the assumption of independent data, future research works will be extended to the conditional performance of different types of control charts for correlated data.
